# Comparison of short-term outcomes of anterolateral supine approach and posterolateral approach for primary total hip arthroplasty: a retrospective study

**DOI:** 10.1186/s10195-021-00570-2

**Published:** 2021-02-27

**Authors:** Taku Ukai, Goro Ebihara, Masahiko Watanabe

**Affiliations:** Department of Orthopaedic Surgery, Surgical Science, Tokai University School of Medicine, 143 Shimokasuya, Isehara, Kanagawa 259-1193 Japan

**Keywords:** Total hip arthroplasty, Postoperative pain, Functional and clinical outcomes, Anterolateral supine approach, Posterolateral approach

## Abstract

**Background:**

This study aims to evaluate postoperative pain and functional and clinical outcomes of anterolateral supine (ALS) and posterolateral (PL) approaches for primary total hip arthroplasty.

**Materials and methods:**

We retrospectively examined the joints of 110 patients who underwent primary total hip arthroplasty (THA). The ALS group was compared with the PL group using the pain visual analog scale (VAS) and narcotic consumption as pain outcomes. Functional outcomes included postoperative range of motion (ROM) of hip flexion, day on which patients could perform straight leg raising (SLR), day on which patients began using a walker or cane, duration of hospital stay, rate of transfer, and strength of hip muscles. Clinical outcomes included pre and postoperative Harris Hip Scores.

**Results:**

No significant differences were found in the pain VAS scores or narcotic consumption between the two groups. The PL group could perform SLR earlier than the ALS group (*P* < 0.01). The ALS group started using a cane earlier (*P* < 0.01) and had a shorter hospital stay (*P* < 0.01) than the PL group. Degrees of active ROM of flexion at postoperative day (POD) 1 were significantly lower in the ALS group than in the PL group (*P* < 0.01). Regarding hip muscle strength, hip flexion was significantly weaker in the ALS group than in the PL group until 1-month POD (*P* < 0.01). External rotation from 2 weeks to 6 months postoperatively was significantly weaker in the PL group than in the ALS group (*P* < 0.01).

**Conclusion:**

The ALS approach was more beneficial than the PL approach because ALS enabled better functional recovery of the strength of external rotation, improved rehabilitation, and involved a shorter hospital stay.

**Level of Evidence:**

Level IV retrospective observational study.

## Introduction

Various approaches are used for total hip arthroplasty (THA) [1–4], and agreement on the best approach remains controversial. The posterolateral (PL) approach is performed extensively worldwide and is the most fundamental approach. However, this approach entails resection of the short external rotators [5]. Thus, studies have reported higher dislocation rates with the PL approach than with other approaches [6–8]. Anterior approaches are divided into the direct anterior approach (DAA) and the anterolateral supine approach (ALS). These approaches have the advantages of being less invasive, having low dislocation rates [9], and resulting in early recovery [10] because both approaches take advantage of the intermuscular plane [11–14]. Although DAA is associated with a lower visual analog scale (VAS) score, longer walking distance, and shorter hospital stay than the posterior approach, it is also associated with lateral femoral cutaneous nerve (LFCN) injury [15, 16]. Previous studies have reported that 23–30% of patients who underwent THA via DAA experienced numbness of the lateral thigh [15, 16]. Compared with DAA, ALS rarely causes LFCN injury and can preserve the anterior capsule, iliofemoral ligament, and conjoint tendon. Preserving these soft tissues is important for hip stability and prevents leg-length discrepancy. However, ALS may cause injury of the inferior branch of the superior gluteal nerve and abductor weakness [17–19].

Although many studies have compared various approaches, aspects of muscle strength other than abduction have rarely been evaluated, and to the best of the authors’ knowledge, no study has assessed pain and functional and clinical outcomes of these approaches simultaneously. Evaluating muscle strength is particularly necessary to assess the advantages of a surgical approach. This study therefore aimed to evaluate and compare short-term results of PL and ALS for primary THA by simultaneously assessing pain and functional and clinical outcomes.

## Materials and methods

### Patients

This study retrospectively examined 110 joints of 110 patients who underwent primary THA between May 2017 and January 2020. All procedures were approved by the ethical committee at the author’s institution (19R-188). One orthopedic surgeon performed all PL procedures, and another orthopedic surgeon performed all ALS procedures. This study only included patients who underwent primary THA and could walk by themselves before operation. Patients who were aged < 40 years, had prior ipsilateral hip surgery, dementia, or mental impairment, or used a walker or wheelchair before operation were excluded.

### Anesthesia and analgesia

All patients underwent THA under general anesthesia maintained with total intravenous propofol. Acetaminophen (1000 mg), fentanyl (100 µg), and metoclopramide (10 mg) were administered at the end of surgery. For postoperative analgesia, oral acetaminophen was administered at a dose of 1800 mg/d starting on the day after surgery. All patients received a cocktail injection comprising 0.2% ropivacaine (150 mg), adrenaline (0.2 mg), methylprednisolone (40 mg), and saline (10 mL) after the placement of the cup and stem component. Additionally, a solution containing 0.2% ropivacaine (150 mg), adrenaline (0.2 mg), and saline (10 mL) was injected intramuscularly (into the m. gluteus medius, m. gluteus maximus, and rotators) and into subcutaneous tissues before wound closure. Wound closure was performed with monofilament suture. Postoperative pain was assessed by a Likert-type scale ranging from 0 to 6 (0, none; 1, very mild; 2, mild; 3, moderate; 4, severe; 5, very severe; 6, intolerable), and severe pain was defined as a score > 4. Patients in severe pain were treated with an intravenous infusion of acetaminophen (500 mg), pentazocine (15 mg), or flurbiprofen (50 mg).

### Surgical procedure

The PL approach was performed in the decubitus position. After the short external rotators were dissected and the femoral heads were removed, a cementless acetabular component (Pinnacle; DePuy Synthes, Leeds, UK) and a cementless proximally porous-coated femoral component (S-ROM; DePuy Synthes, Warsaw, IN, USA) were placed. Head sizes of 32 mm were used for all patients. After the placement of the acetabular and femoral components, the short external rotators were repaired by suturing with the greater trochanter. The posterior capsule was also sutured. For all patients, weight-bearing was allowed on the day after surgery as tolerated.

The ALS approach was performed in the supine position, and an image intensifier was used. A cementless acetabular component (Continium; Zimmer Biomet, Warsaw, IN, USA) and cementless proximally porous-coated femoral component (Fitmore; Zimmer Biomet, Winterthur, Switzerland) were placed. The vertical iliofemoral ligament, conjoint tendon, and anterior capsule were preserved. Similar to the PL group, weight-bearing for the ALS group was allowed on the day after surgery as tolerated.

### Measurement of muscle strength

The muscle strengths of hip flexion, extension, abduction, adduction, internal rotation, and external rotation were quantified by using a handheld dynamometer (Anima Co., µ-TasF1, Tokyo, Japan). To standardize the measurement, only one person performed muscle strength measurement of the hip. The measurement was performed in a hospital or an examination room, and we performed the measurement before rehabilitation to avoid fatigue. During the measurement of hip flexion, abduction, and adduction, patients were placed in the supine position with the hips and knees in a neutral position. Muscle strengths during hip internal rotation and external rotation were measured with the patients seated and the hips and knees in 90° flexion. For hip extension, the patients were placed in the prone position with the hips and knees in a neutral position. Each subject performed two trials for all examinations, and the highest peak force was used for analysis. The peak force was normalized to each patient’s body weight (N/kg). We measured the muscle strength on the day of admission and at 2 weeks, 1 month, 3 months, and 6 months after the operation.

### Implant alignment

Postoperative cup inclination, cup anteversion, and stem anteversion were evaluated by using computer tomography. Cup inclination in the coronal plane was measured between the transverse axis of the cup and the inter-teardrop line. Cup anteversion was measured in the axial plane as the angle between the transverse cup axis and the sagittal plane. Stem anteversion was measured in the axial plane relative to the posterior bicondylar plane of the femur [20].

### Clinical outcome

The Harris Hip Score (HHS) [21] was used to evaluate the preoperative and postoperative clinical outcomes. The HHS was evaluated on the day of admission and at 1 month, 3 months, and 6 months after the operation.

### Evaluation

Preoperative mental wellness was evaluated using the Japanese Orthopedic Association Hip Disease Evaluation Questionnaire (JHEQ) [22]. JHEQ is a self-report questionnaire and consists of three categories: pain, activities of daily living, and mental wellness. Each question is scored using a Likert-type scale (0 points, strongly agree; 1 point, agree; 2 points, uncertain; 3 points, disagree; 4 points, strongly disagree). The mental wellness score of JHEQ ranges from 0 to 28 points, with 0 points indicating the lowest level of mental wellness and 28 points indicating the highest level of mental wellness [22] (Table 1).

**Table 1:** Mental category of the JHEQ

Question
Because of my hip-joint disease, I feel dissatisfied with my health
My hip-joint condition deeply affects my well-being
Because of my hip-joint disease, I sometimes feel down
Because of my hip-joint disease, it is difficult to actively undertake various things
Because of my hip-joint disease, I notice how others look at me
Because of my hip-joint pain, sometimes participation in local events and neighborhood relationships do not go smoothly for me
Because of my hip-joint disease, I sometimes quarrel with people

JHEQ: Japanese Orthopedic Association Hip Disease Evaluation Questionnaire

Pain was evaluated using VAS (0 mm = no pain; 100 mm = worst imaginable pain) and narcotic consumption. The VAS scores were recorded while patients were at rest in the morning. Pain VAS evaluation was conducted from the day of the surgery (1 h after surgery) to 7 days after surgery. Narcotic consumption was recorded until postoperative day (POD) 3. Functional outcome was evaluated using the following information: the active range of motion (ROM) of hip flexion, day on which patients were able to perform straight leg raising (SLR), and day on which patients began using a walker or a cane. ROM and SLR were evaluated in the morning. When ROM was evaluated in a supine position, patients attempted to flex their operated hips independently. The SLR test was also conducted in a supine position, and we considered patients’ ability to perform SLR as their ability to keep their lower limbs raised for at least 3 s. Furthermore, we evaluated the length of hospital stay and the rate of transfer. Patients were allowed discharge when they were able to ascend and descend stairs with a cane independently. Patients who were not discharged home until POD 28 were transferred to other hospitals.

### Statistical analysis

Student’s *t* test was used to evaluate age, body mass index (BMI), operation time, VAS, the mental wellness score, cup inclination, cup anteversion, stem anteversion, narcotic consumption, muscle strength (i.e., hip flexion, extension, abduction, adduction, internal rotation, and external rotation), ROM, day on which patients were able to perform SLR, day on which patients began using a walker or a cane, duration of hospital stay, and HHS between the PL and ALS groups. Fisher’s exact test was also used to evaluate distribution of sex, diagnosis, and rate of transfer to other hospitals. All tests were performed with a significance level of *P* < 0.05. Analyses were performed using the SPSS statistical software (version 26; IBM Corp., Armonk, NY, USA).

## Results

The PL approach was performed on 63 patients (13 male, 50 female) with a mean age of 65 ± 11.8 years. The ALS approach was performed on 37 patients (3 male, 34 female) with a mean age of 66.7 ± 11.1 years.

**Fig. 1:**
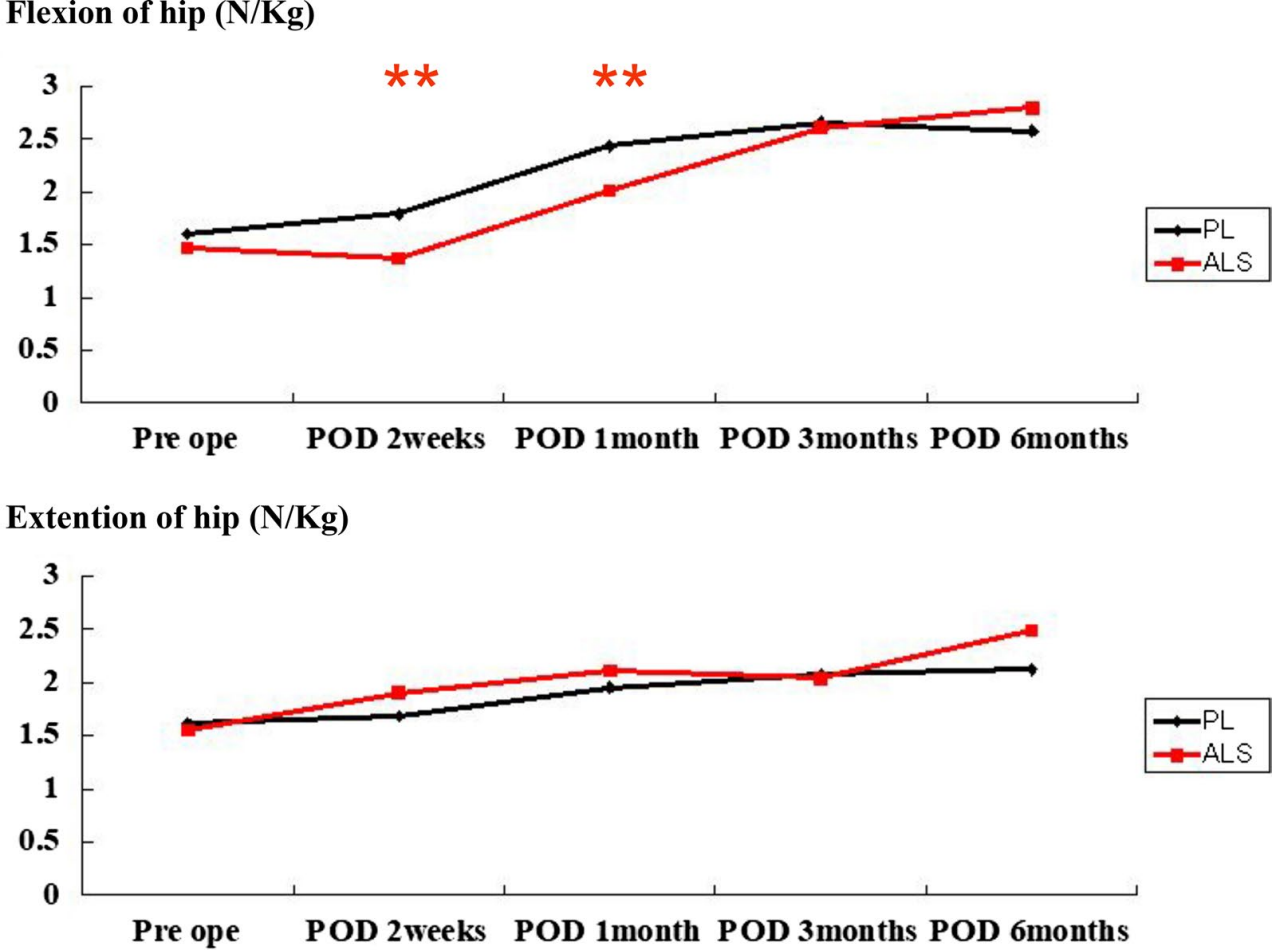
Evaluation of muscle strength of hip flexion and extension. Hip flexion was significantly weaker in the ALS group than in the PL group until 1 month after operation. ALS: anterolateral supine; PL: posterolateral; POD: postoperative day. **Indicates significance at *P* < 0.01

**Fig. 2:**
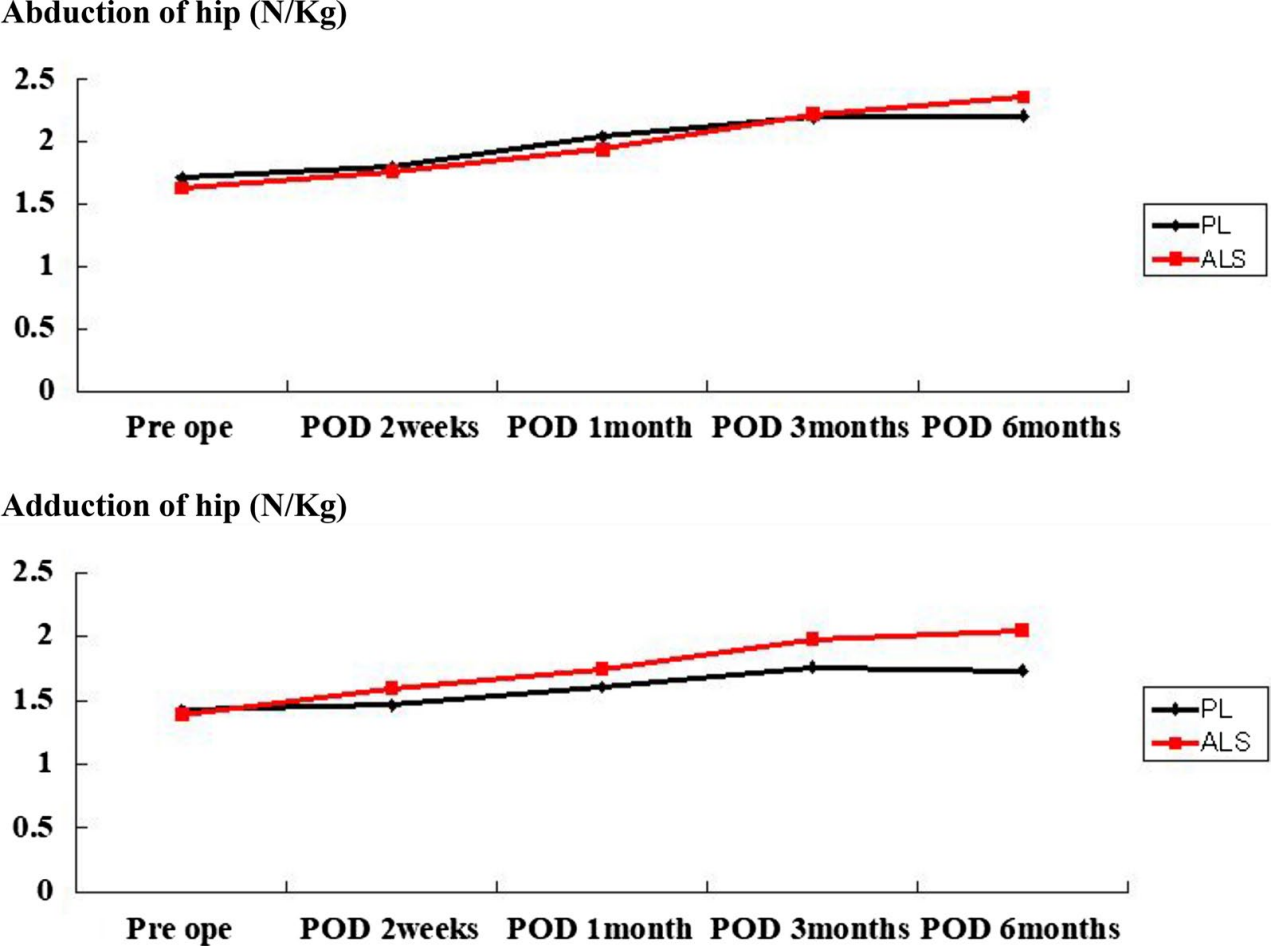
Evaluation of muscle strength of hip abduction and adduction. No significant difference was found in hip abduction or adduction between the two groups. ALS: anterolateral supine; PL: posterolateral; POD: postoperative day

**Fig. 3:**
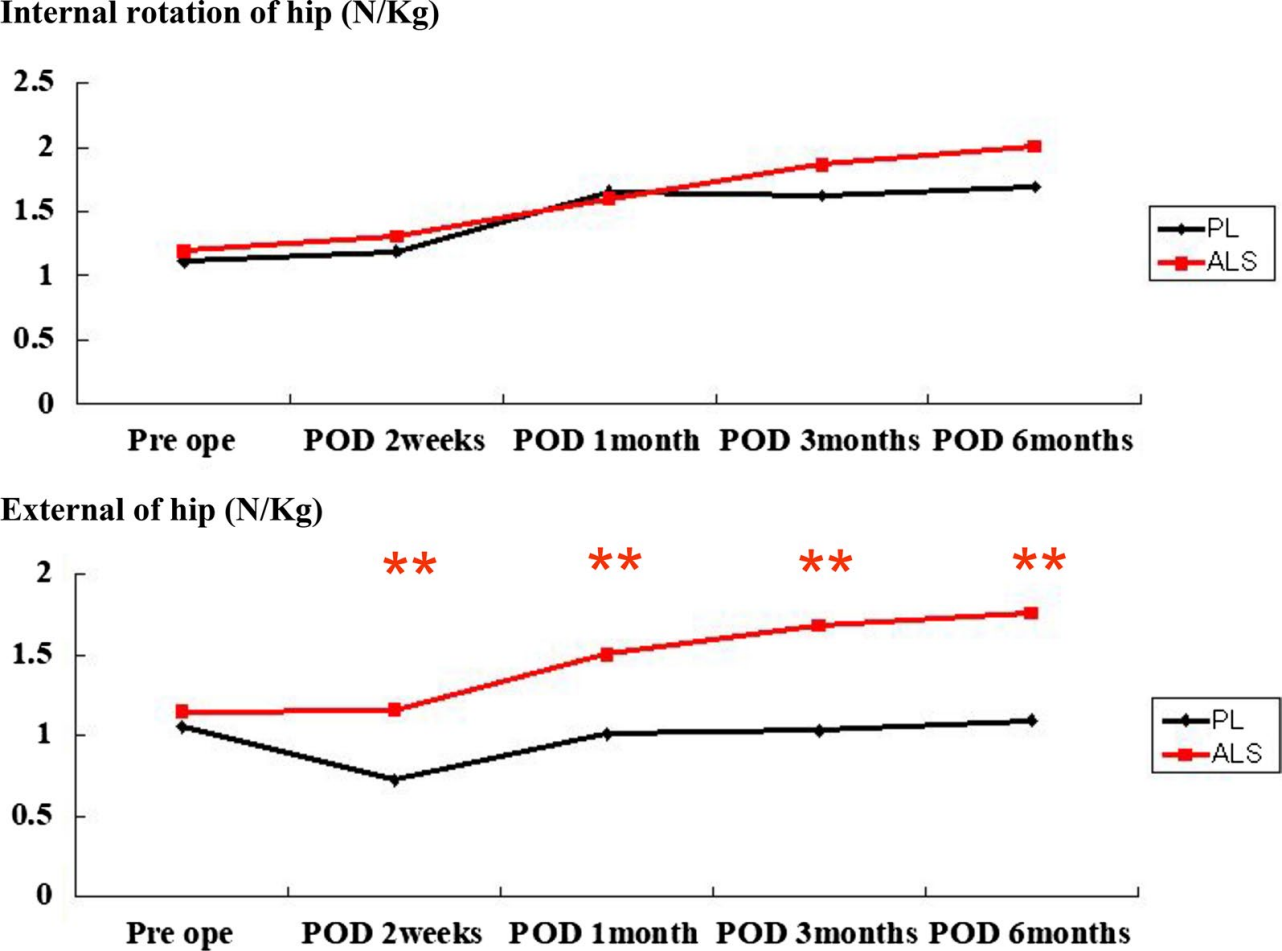
Evaluation of muscle strength of hip internal and external rotation. From 2 weeks to 6 months postoperatively, hip external rotation strength was significantly lower in the PL group than in the ALS group. ALS, anterolateral supine; PL, posterolateral; POD, postoperative day. **Indicates significance at *P* < 0.01

There were no significant differences in cup inclination, cup anteversion, or stem anteversion between the two approaches (Table 2). Operation time in the ALS group was significantly longer than that in the PL group (*P* = 0.00) (Table 3). No significant difference was found between the two groups at any point of VAS and with regard to number of narcotics consumed (Table 4). Degrees of active ROM of flexion at POD 1 were significantly lower in the ALS group than in the PL group (*P* < 0.01). The PL group was able to perform SLR earlier than the ALS group (*P* < 0.01). No significant difference was found regarding the day on which patients began using a walker. However, the day on which patients began using a cane was significantly earlier (*P* < 0.01) and hospital stay was significantly shorter (*P* < 0.01) in the ALS group than in the PL group. No significant difference was found regarding rate of transfer (Table 5). Until 1 month after operation, hip flexion was significantly weaker in the ALS group than in the PL group (*P* < 0.01) (Fig. 1). No significant difference was found in hip abduction or adduction between the two groups (Fig. 2). From 2 weeks to 6 months postoperatively, hip external rotation strength was significantly lower in the PL group than in the ALS group (*P* < 0.01) (Fig. 3). No significant differences were found in the preoperative or postoperative HHS between the two groups.

**Table 2:** Postoperative alignment of implants in the PL and ALS groups

Implant alignments	PL	ALS	*P* value
Cup inclination (°)	39.4 ± 7	41.7 ± 6.3	0.62
Cup anteversion (°)	24.9 ± 11.9	26.3 ± 14.7	0.27
Stem anteversion (°)	30.4 ± 10.6	30.8 ± 15.2	0.98

ALS: anterolateral supine; PL: posterolateral

**Table 3:** Demographic data of patients in the PL and ALS groups

Patient characteristics	PL	ALS	*P* value
Age (years)	65 ± 11.8	66.7 ± 11.1	0.49
Sex	Male to female (13:50)	Male to female (3:34)	0.06
Diagnosis	OA 55, ION 7, RDC 1	OA 25, ION 7, RDC 1, RA 4	0.11
BMI	23.9 ± 2.9	22.4 ± 3.6	0.06
Preoperative ROM (degrees)	87.7 ± 21.7	84.5 ± 23.4	0.49
Operation time (minutes)	58.9 ± 15.7	112 ± 29.5	0
Preoperative VAS score	84.7 ± 14.3	85.3 ± 18.1	0.86
Preoperative mental wellness score	7.4 ± 5.2	7.7 ± 6.1	0.82
Preoperative muscle strength (N/kg)			
Flexion	1.6 ± 0.8	1.5 ± 0.6	0.4
Extension	1.6 ± 0.5	1.6 ± 0.5	0.53
Abduction	1.7 ± 0.5	1.6 ± 0.6	0.5
Adduction	1.4 ± 0.5	1.4 ± 0.4	0.67
Internal rotation	1.1 ± 0.4	1.2 ± 0.4	0.35
External rotation	1.1 ± 0.4	1.1 ± 0.4	0.27

ALS: anterolateral supine; BMI: body mass index; ION: idiopathic osteonecrosis; OA: osteoarthritis; PL: posterolateral; RA: rheumatoid arthritis; RDC: rapidly destructive coxopathy; VAS: visual analog scale

**Table 4:** Postoperative pain (VAS scale) and narcotic consumption of patients in the PL and ALS groups

VAS at different PODs and number of narcotics consumed	PL	ALS	*P* value
VAS (POD 0)	46.9 ± 28.1	51.2 ± 28.4	0.47
POD 1	40.5 ± 26.5	35.3 ± 28	0.36
POD 2	35.8 ± 26.1	32.3 ± 25.9	0.53
POD 3	24.4 ± 23.5	25.3 ± 26.1	0.85
POD 4	20.5 ± 22.2	18.2 ± 21.7	0.63
POD 5	17.4 ± 21	17.5 ± 19.3	0.97
POD 6	12.4 ± 16.9	16.5 ± 19.2	0.27
POD 7	11.7 ± 17.2	16 ± 18.4	0.25
Narcotic consumption (numbers)	1.7 ± 1.8	1.8 ± 2.1	0.51

ALS: anterolateral supine; PL: posterolateral; POD: postoperative day; VAS: visual analog scale

**Table 5:** Postoperative functional evaluation of patients in the PL and ALS groups

Factors for functional evaluation	PL	ALS	*P* value
ROM (POD 1) (degrees)	55.7 ± 19.8	42.6 ± 18.5	0.001
POD 2	61.7 ± 19	55.4 ± 20.6	0.13
POD 3	70 ± 17.6	63.2 ± 26.5	0.13
POD 4	78.4 ± 17.5	72.7 ± 25.9	0.19
POD 5	82.8 ± 17	77.8 ± 21.8	0.22
POD 6	84.8 ± 17.1	78.6 ± 19.4	0.1
POD 7	86.1 ± 18.4	81.2 ± 18.7	0.21
SLR (days until achievement)	2.5 ± 2.9	4.3 ± 3.4	0.006
Walker (days until achievement)	4.4 ± 2.3	3.9 ± 2.1	0.31
Cane (days until achievement)	9.1 ± 3.5	7.1 ± 2.6	0.004
Hospital stay (days)	18.6 ± 4.1	15.5 ± 3.6	0.001
Rate of transfer	4/63	3/34	1.0

ALS: anterolateral supine; PL: posterolateral; POD: postoperative day; ROM: range of motion; SLR: straight leg raising

## Discussion

In this study, we compared the outcomes of the PL and ALS approaches among patients who underwent THA. We found no significant difference in pain outcome between the two groups. For functional evaluation, the PL group was able to perform SLR significantly earlier than the ALS group. Until 1 month postoperatively, the strength of hip flexion was significantly weaker in the ALS group than in the PL group. However, the day on which patients began using a cane was significantly earlier, hospital stay was significantly shorter, and from 2 weeks to 6 months postoperatively, the strength of hip external rotation was significantly higher in the ALS group than in the PL group.

Various approaches are used for THA. Each approach has both advantages and disadvantages. Although the PL approach is used extensively worldwide, it has a high risk of dislocation compared with other approaches [23]. On the contrary, the ALS approach has been reported to lead to a low dislocation rate, less postoperative pain, better functioning of the abductor muscle, and a shorter hospital stay [2, 6, 24–26]. However, this approach is correlated with the damage to the femoral shaft and malalignment of the femoral component [27].

With regard to postoperative pain, Wang et al. compared DAA with PL and found that the DAA group reported significantly lower pain intensity at 24, 48, and 72 h after surgery [28]. In the present study, no significant difference was found in postoperative pain VAS scores between the ALS group and the PL group. We initially considered that, when we conducted the ALS approach, we may have injured the gluteus medius muscle, which could have affected the pain VAS score because the ALS approach has been reported to cause superior gluteal nerve and gluteus medius muscle injuries [17–19]. However, the measurement of the muscle strength during hip abduction revealed no significant difference between the ALS group and the PL group (Fig. 2). In this study, operation time was significantly longer in the ALS group than in the PL group. Even if conventional, the ALS approach tends to require a longer operation time than the PL approach because the PL approach is one of the most basic approaches. On the other hand, the ALS approach is more difficult to perform than the PL approach, particularly with regard to stem insertion.

The iliofemoral ligament is the strongest ligament of the hip capsule and works to resist the external rotation and extension of the hip [29, 30]. Although we initially resected the anterior capsule and iliofemoral ligament, we preserved them in this study to enforce stability of the hip and avoid leg-length discrepancy. However, preserving the iliofemoral ligament entails the restriction of the surgical field and restriction of the elevation of the femur. Due to that, this method tends to require more time for retracting muscles of the anterior hip than the conventional ALS approach. This means that retracting the muscles of the anterior hip may cause swelling of the anterior hip muscles and increase the pain VAS score, especially during the early post-THA period. In fact, patients in the ALS group were able to perform SLR significantly later than patients in the PL group (Table 5).

With regard to the functional outcome, Higgins et al. compared the DAA group and the PL group by assessing estimated blood loss, intraoperative fractures, length of hospital stay, and likelihood of discharge and reported that the DAA group had better outcomes related to length of stay and dislocations [4]. Wang et al. also compared the DAA group and the PL group by assessing the pain VAS scores, incision length, operation time, blood loss, length of hospital stay, and complications, and they reported that the DAA approach was associated with early functional recovery compared with the PL approach [28]. However, they did not assess muscle strength, ROM, or functional outcomes (e.g., SLR and use of a walker or cane). Although these factors are necessary for assessing functional recovery, they are rarely evaluated. Compared with the PL approach, the DAA and ALS approaches are considered less invasive because they do not involve the cutting of any muscles. Nevertheless, the extent and duration of motor weakness is still unclear. Although our findings revealed that the ALS approach was correlated with a delay in SLR and weakness of the anterior hip muscles until 1 month postoperatively, we believe that the ALS approach leads to better functional outcomes than the PL approach because weakness in the hip external rotators persisted for up to 6 months after surgery in the PL group. In addition, patients in the ALS group began using a cane earlier than those in the PL group and experienced shorter hospital stays.

Several limitations in this study should be noted. First, this study had a retrospective design. Second, the ALS and PL approaches were not performed by the same surgeons, which may have affected functional outcomes. However, they each had extensive experience as a hip surgeon (> 10 years, > 100 cases). Additionally, both surgeons participated in all operations, and surgical procedures were unified. Third, we did not assess motion pain after rehabilitation. We did initially attempt to assess motion pain after THA, but standardizing the assessment of motion pain proved to be too difficult because ROM of the hip and progress of rehabilitation varied according to each individual. For instance, the degree of motion pain after gait training compared with that after rehabilitation in bed are quite different. Fourth, muscle strength was measured by only one person who was not blinded and there was no mediator, which may have biased the results. However, although it is possible for the measurement of muscle strength to be highly variable, the tester has had sufficient experience with a handheld dynamometer (> 5 years and > 100 patients). Fifth, each approach was performed with different implants, and only the ALS approach used intraoperative image intensifiers. Although there is no obvious evidence that intraoperative image intensifiers or different implant systems directly influence postoperative pain, rehabilitation, or muscle strength, different implant systems can influence the malalignment of implants, and some studies have reported that implant alignment differed by approach [31, 32]. There is also a possibility that implant alignment affects muscle tension and gait, as asserted by Tsai et al. who reported that cup anteversion influenced the hip kinematics during gait [33]. However, there were no significant differences in cup inclination, cup anteversion, or stem anteversion between the two approaches in the present study (Table 2). Thus, we do not believe that intraoperative image intensifiers or different implant systems affected the results of this study. Finally, the length of operation time was significantly different between the ALS and PL groups, which may have affected pain VAS scores and functional outcomes. In the future, we plan to investigate the short-term results between the ALS and PL approaches by conducting a randomized controlled study.

In conclusion, the ALS approach was correlated with weakness of the anterior hip muscles during the early post-THA period. However, functional outcomes of the ALS group were better than those of the PL group because ALS enabled a better functional recovery of the strength of external rotation, improved rehabilitation, and resulted in shorter hospital stays.

## Data Availability

Not applicable.

## Abbreviations

ALS: Anterolateral supine
BMI: Body mass index
DAA: Direct anterior approach
JHEQ: Japanese Orthopedic Association Hip Disease Evaluation Questionnaire
HHS: Harris Hip Score
LFCN: Lateral femoral cutaneous nerve
PL: Posterolateral
POD: Postoperative day
ROM: Range of motion
SLR: Straight leg raising
THA: Total hip arthroplasty
VAS: Visual analog scale
